# An open source device for operant licking in rats

**DOI:** 10.7717/peerj.2981

**Published:** 2017-02-14

**Authors:** Matthew Longley, Ethan L. Willis, Cindy X. Tay, Hao Chen

**Affiliations:** 1 Undergraduate Program, University of Memphis, Memphis, TN, United States; 2 Maters’ Program, Department of Bioinformatics, University of Memphis, Memphis, TN, United States; 3 Undergraduate Program, Duke University, Durham, NC, United States; 4 Department of Pharmacology, University of Tennessee Health Science Center, Memphis, TN, United States

**Keywords:** Operant behavior, Behavior testing device, Open source, Single board computer, Licking response, Environment variables

## Abstract

We created an easy-to-use device for operant licking experiments and another device that records environmental variables. Both devices use the Raspberry Pi computer to obtain data from multiple input devices (e.g., radio frequency identification tag readers, touch and motion sensors, environmental sensors) and activate output devices (e.g., LED lights, syringe pumps) as needed. Data gathered from these devices are stored locally on the computer but can be automatically transferred to a remote server via a wireless network. We tested the operant device by training rats to obtain either sucrose or water under the control of a fixed ratio, a variable ratio, or a progressive ratio reinforcement schedule. The lick data demonstrated that the device has sufficient precision and time resolution to record the fast licking behavior of rats. Data from the environment monitoring device also showed reliable measurements. By providing the source code and 3D design under an open source license, we believe these examples will stimulate innovation in behavioral studies. The source code can be found at http://github.com/chen42/openbehavior.

## Introduction

Quantitative measurement of rodent behavior is one of the cornerstones of neuroscience. Traditionally, neuroscientists have favored the approach of vastly reducing the complexity of the experimental paradigm. However, more recently, many scientists have realized that in order to better grasp the complexity of behavior, data sets capturing many more internal and environmental variables and with much higher time and spatial resolution are required (Gomez-Marin et al., 2014).

Recent generations of single-board computers are the size of a credit card and yet are as powerful as desktop computers from a few years ago. Furthermore, numerous sensors are available to be connected to these computers. These sensors can be used to monitor a wide variety of environmental variables as well as animal behaviors. Therefore, there exists an opportunity to exploit these readily available electronic and computing resources for measuring multi-dimensional behavioral data.

**Figure 1:**
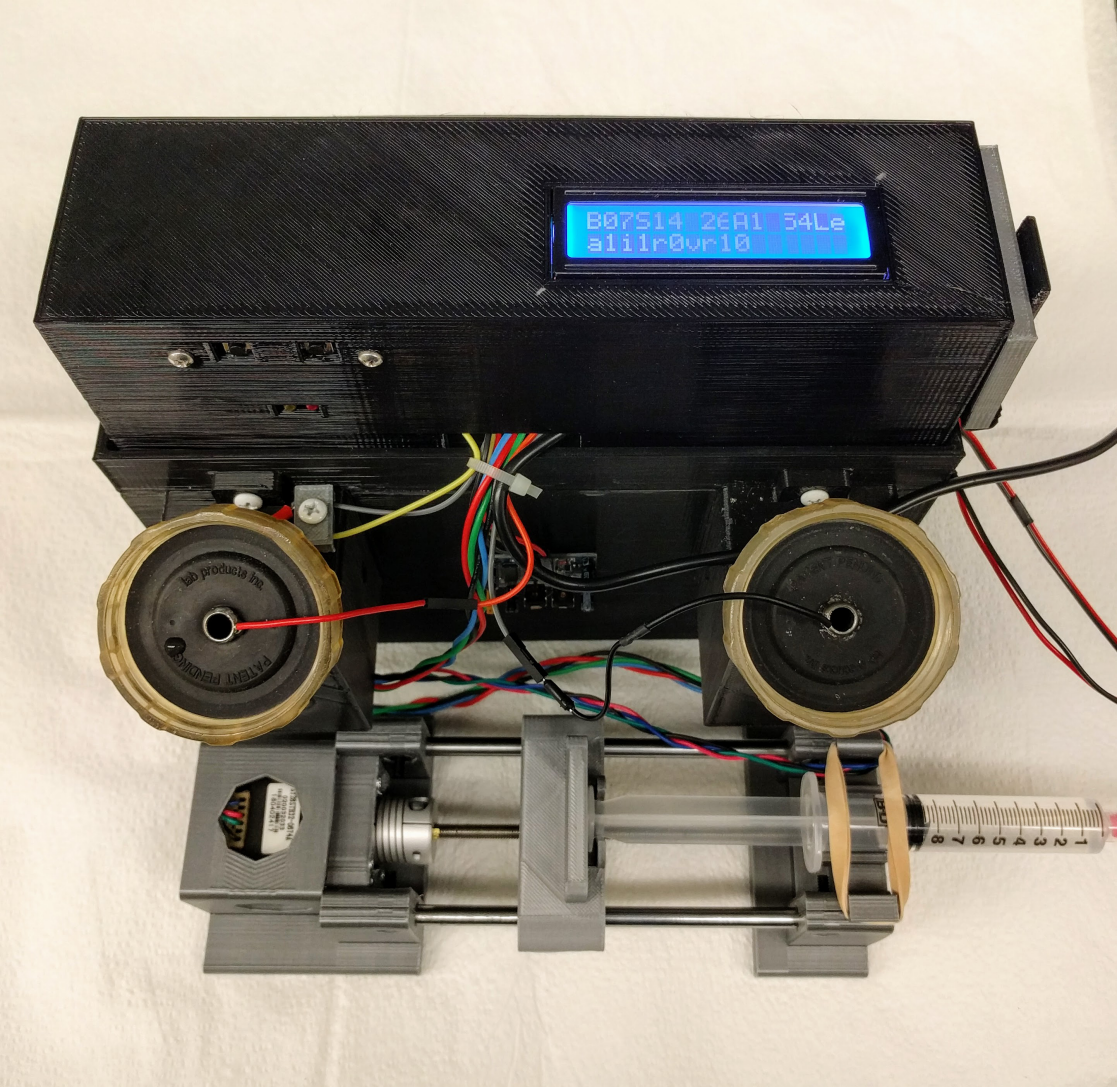
The operant licking device. The 3D printed frame holds two lick spouts that are connected to a touch sensor. The antenna of an RFID reader (grey) is stored in its holder. The syringe pump delivers a fixed amount of solution when the number of licks completes a reinforcement schedule. The position of the pump can be manually adjusted using the two buttons on the back of the frame. More photos are available in our GitHub repository.

Here, we describe a project where a Raspberry Pi^®^ single-board computer was used to study operant licking in rats. Operant training in animals allows them to associate a response (e.g., peck a spot with the beak, press a lever, or lick a spout) with a reward (e.g., food, water, or drugs), as well as reward-associated cues (e.g., a light). Operant conditioning is used to study a wide variety of behaviors, especially those related to the function of the reward system, such as food consumption or drug abuse. We also used a motion sensor to track the activity of the rat and a radio-frequency identification (RFID) tag to track the identity of the rat (Fig. 1). In addition, we also designed an environmental sensor set that monitors the temperature, humidity, barometric pressure, and ambient light levels.

## Methods

### The operant licking device

This system uses a capacitive touch sensor (MPR121; Adafruit) connected to the drinking spouts to record the licking behavior of rodents. When the number of licks on a spout meets a predetermined criteria, a syringe pump is triggered to deliver a fixed amount of solution to that spout. A visual cue (an LED) is turned on every time the solution is delivered. A motion sensor is used to record the activity of the rodent. An RFID reader is used to read the glass ID tag embedded in the animal. All the data are recorded locally on the storage media (a Secure Digital card) and are transferred to a remote server via wireless internet connection at the end of the sessions using the rsync command. All electronic devices are installed in a 3D printed frame and can be placed in a standard rat cage. The syringe pump can be placed on top of the wire grid of the cage. The design source files used for 3D printing, software, and instructions for assembly are available in our github repository (https://github.com/chen42/openbehavior) under the Creative Commons Attribution NonCommercial license (Version 3.0).

#### Computer

**Figure 2:**
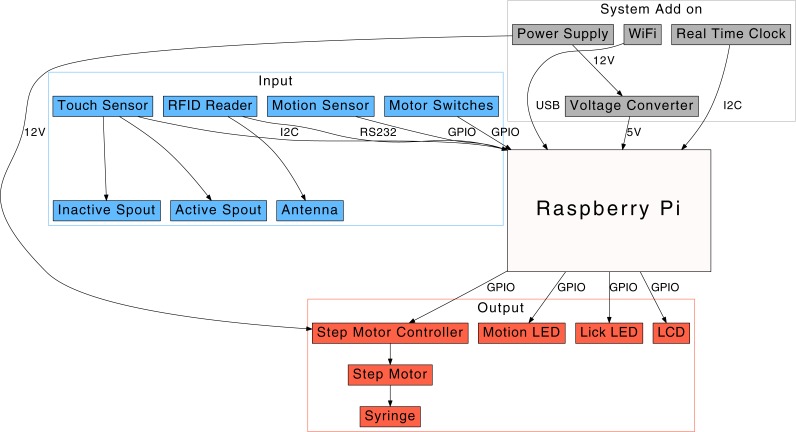
A diagram of the operant licking system. A touch sensor is used to measure the number of licks by a rat on a spout. When the number of licks reaches the criterion, the Raspberry Pi computer advances a step motor, which in turn pushes a syringe to deliver a drop of solution. A motion sensor is used to record the movement of the rat.

We used the Raspberry Pi (model 2B; RPi) single-board computer. The computer uses a Broadcom BCM2836 Arm v7 quad core processor (900 MHz) and has 1 GB RAM. A total of 40 pins are available on a header for user expansion, including 17 GPIO pins, an I2C connection, and a serial line. Although sufficiently powerful, the RPi 2B is missing a few key components. Therefore, we added a WiFi module (EW-7811Un; Edimax) and a real time clock (DS1307; JBtek). The clock is connected to the RPi via an I2C interface and is needed to maintain the system time when network connectivity is not available. The more recent Raspberry Pi 3 model, which includes a WiFi module, can also be used after its bluetooth interface is disabled (a script is provided in our GitHub repository). The system power is provided by a 12 V AC-DC converter (Binzet, regulated, 2 Amp). A DC-DC voltage step down module (LM2596; Texas Instruments) is used to provide 5 V power to the RPi. A diagram of the peripheral components connected to the RPi is shown in Fig. 2.

#### Input devices

The key component of the system is the MPR121 capacitive touch sensor (Adafruit), which communicates with the RPi via the I2C protocol. We used only two of the 12 channels of this sensor. Each channel was connected to one drinking spout to monitor the licking behavior. A passive infrared motion detector (HC-SR501; Adafruit) was used to monitor the activity of the animal. Located within this detector are two infrared sensors. The differences in infrared signals between these two dectors are used as a measure of activity. We have observed that the activity of the rat in either end of a standard rat box measuring 17 in (L) × 8 in (W) reliably triggered the sensor (based on the LED motion indicator), and no signal was generated when the rat was resting . We also connected a RFID reader (RDM6300; Itead) to the RPi. This reader can detect low frequency (125 kHz) glass RFID tags with EM4100 standard (HeHeng-RFID via AliExpress.com, EM4100). These glass tags can be implanted under the skin of the rats to provide a unique identification code for each rat. Lastly, we installed two push buttons to provide bidirectional manual control of the step motor. This allows the position of the motor to be adjusted when loading the syringe.

#### Output devices

The main output device is a syringe pump modified from the design provided by Wijnen et al. (2014). A step motor controller (model A4988, with a heat sink; Elegoo) is used to advance a step motor (Pololu, NEMA 11). The motor is installed in a 3D printed frame, which also has a holder for a 10 ml syringe. The motor advances the syringe to deliver one drop (calibrated to be 64.5 ± 5.75 µl, mean and standard deviation) of solution to the tip of the spout (via a polyethylene tubing) each time it is activated. Three LEDs are connected to the device. One serves as a cue light (Fulight^®^ clear, 5 mm in diameter, 6,000 mcd at 20 mA, 60° viewing angel) and is installed on top of the active spout. The other two LEDs are indicators of licking or locomotor activity. Lastly, an LCD (16 × 2 characters) is connected to the GPIO pins. The LCD provides information about the system before, during, and after the test sessions.

#### Software and reinforcement schedules

Our software is written in the Python programming language and runs on the Raspbian Linux operating system (Jessie, released on 05-27-2016). Libraries for the touch sensor (Adafruit Python MPR 121) and the LCD (Adafruit Python CharLCD) were provided by the manufacture. We adjusted the default thresholds of the MPR 121 library to touch = 36 and release = 18 (in the self.set_thresholds function) before system wide installation. This adjustment was necessary to eliminate the false touch signal when the sensor was connected to the spouts. We observed 0 count of touching event when the spout was left untouched for three hours after this adjustment.

The main program is automatically launched at system start up. Once the program is loaded, two push buttons can be used to adjust the position of pump for loading the syringe. The system then awaits input from the RFID scanner. The session timer starts when the technician scans the glass RFID tag embedded in the rat. The touch sensor records the timing of licking from two spouts, designated as the active and the inactive spout, respectively. We implemented three reinforcement schedules. When the fixed ratio (FR) schedule is used, a reward (i.e., fluid delivered to the active spout) is delivered when a predetermined number of licks (e.g., 10) is recorded. A variable ratio (VR) schedule delivers the reward after a randomly chosen number of licks, with a predetermined average (e.g., 10). When controlled by a progressive ratio schedule, the number of licks required to obtain each subsequent reward is increased until the animal fails to obtain a reward within a given time period. A time out period, when the number of licks is recorded but has no programmed consequence, is enforced after each reward. Because there is no conventional input device present, such as a keyboard or a pointing device, we use a few specific RFIDs to switch between these reinforcement schedules (i.e., scanning one particular RFID tag will load the progressive ratio schedule).

A cue light located above the active spout (characteristics described above) is turned on for a fixed time (e.g., 5 s) after each reward. The number of licks on the inactive spout is recorded throughout the session but has no programmed consequence. Data from the motion sensor is recorded using a separate Python program. In addition to the timing and type of each event (i.e., lick, movement), the start and finish times are recorded in the data files. The LCD is used throughout the session to provide system status and real time data. The Linux program rsync is used to automatically transfer data files to a remote server upon the completion of each session.

### The environmental variable monitoring device

The standalone environmental variable monitoring device also uses a RPi. Four sensors are connected to the RPi via the I2C communication protocol. These are the TSL2561 for ambient light, HTU21D-F for humidity, and BMP085 for barometric pressure. Both the HTU21D-F and the BMP085 contain a temperature sensor. We recorded the average reading from these two sensors. The Python libraries provided by Adafruit for these specific products are used to obtain data. The logging program was excecuted once every 10 min as a cron job. The Linux rsync program is used to transfer the collected data to a remote server automatically. The Python programs are available in our github repository listed above.

### Procedure for implanting RFID

RFID tags and matching insertion needles (HeHeng-RFID, via http://AliExpress.com) were sterilized by soaking in Cidex Plus (Advanced sterilization products) overnight, followed by washing in 70% ethanol and sterile saline. The injection procedure was similar to that of a subcuetaneous injection. Briefly, the skin around the nape of the neck was wiped with alcohol before a rat was restrained in a plastic wrap. The needle containing a RFID was then rapidly inserted under the skin and the RFID injected.

### Operant licking for sucrose or water

Ten female rats purchased from Harlan Laboratory were used. Animals were housed in a reverse light-cycled room (lights off at 9:00 AM and on at 9:00 PM). Food and water were provided *ad libitum*. These rats were tested under a FR10, a VR10, and a PR schedule to obtain a 10% sucrose solution (*n* = 5) or water (*n* = 5). The FR10 and VR10 sessions lasted 1 h. The PR session ended 10 min after the last lick on the active spout. These tests were run in a dark room with all lights turned off. All procedures were conducted in accordance with the NIH Guidelines Concerning the Care and Use of Laboratory Animals and were approved by the Institutional Animal Care and Use Committee of the University of Tennessee Health Science Center.

### Statistical analysis

Data are presented as mean ± standard error. Student’s *t*-tests were used to analyze the difference between the licks on the active vs. the inactive spouts. Statistical significance was assigned for *p* < 0.05 . The R statistical analysis language was used for data analysis and plotting.

**Figure 3:**
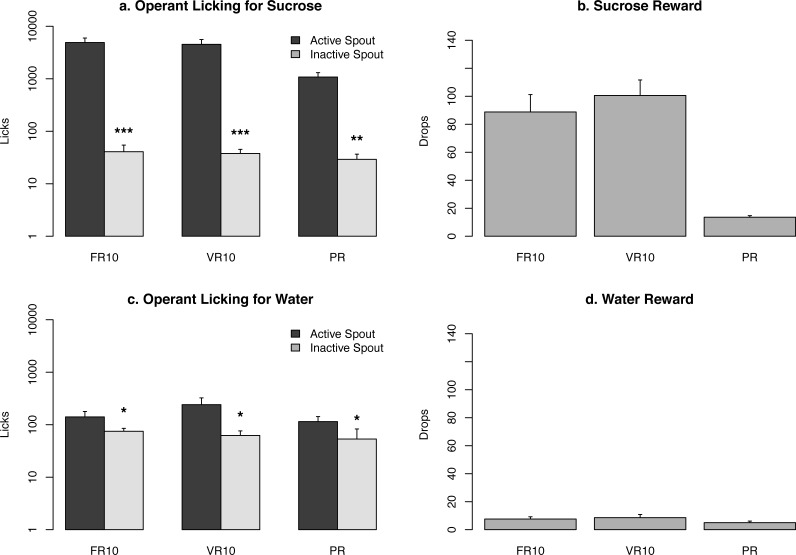
Summary of lick responses and rewards. Ten rats were tested using the operant licking devices to obtain a sucrose solution (10%, *n* = 5) or water (*n* = 5) under the control of a fixed ratio 10 (FR10), a variable ratio 10 (VR10), or a progressive ratio schedule (VR). A logarithmic scale is used for the *Y*-axis for (A and C). *p < 0.05 , ***p < 0.001 , compared to the active spout.

## Results

### Operant licking response for sucrose

We tested two groups of rats for operant licking in five devices assembled as describe above. Rats obtained either a sucrose solution (10%, *n* = 5) or water (*n* = 5). Each rat was tested using the FR10, VR10, and PR reinforcement schedules. The number of licks and rewards for each data set are plotted in Fig. 3. Rats licked 4,902 ± 1, 076, 4,525 ± 1, 072, and 1,081 ± 227 times on the active spouts when tested on the FR10, VR10, and PR reinforcement schedules, and received 89 ± 12, 101 ± 11, 14 ± 1 drops (60 µl) of sucrose solution, respectively. In contrast, rats licked 141 ± 38, 241 ± 84, and 115 ± 28 times on the active spouts when tested on the FR10, VR10, and PR reinforcement schedules, and received 8 ± 2, 9 ± 2, 5 ± 1 drops of water, respectively. The number of licks on the active spout was significantly greater than those on the inactive spouts when sucrose or water was provided (Fig. 3). These results are very similar to those reported by Sclafani & Ackroff (2003), indicating that the devices provided reliable data.

**Figure 4:**
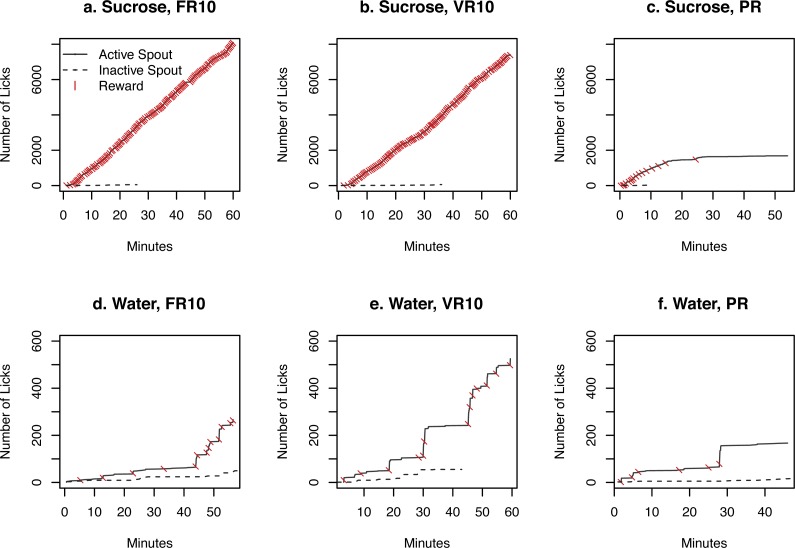
Time course of licks and rewards. The cumulative number of licks by one rat on the active and inactive spouts as well as rewards (i.e., 60 µl sucrose solution or water) earned during one test session are shown. A rat licked continuously on the active spouts for sucrose when the FR10 or VR10 schedule was used. The rate of licking slowed down dramatically under the PR ratio, where the workload for each subsequent reward increased rapidly. The rate of licking was much lower when water was provided.

The time course of licks on the two spouts as well as rewards earned by the rat with the highest number of licks in each of the six test conditions was plotted in Fig. 4. Rats licked almost continuously on the active spouts for the entire 60 min testing period for sucrose when the FR10 or the VR10 schedule was used. The rate of licking was much lower when the PR schedule was used. Although they sampled the inactive spout initially, licking activity became exclusive for the active spout near the end of each of the sessions. In contrast, when water was provided, the licks were much sparser, with large time lags between receiving rewards. Thus, these data better illustrate the number of licks between rewards. The number of licks was not even when the FR10 schedule was used. This is because rats continue to lick on the active spout during the 20 s time out period after the reward was delivered.

**Figure 5:**
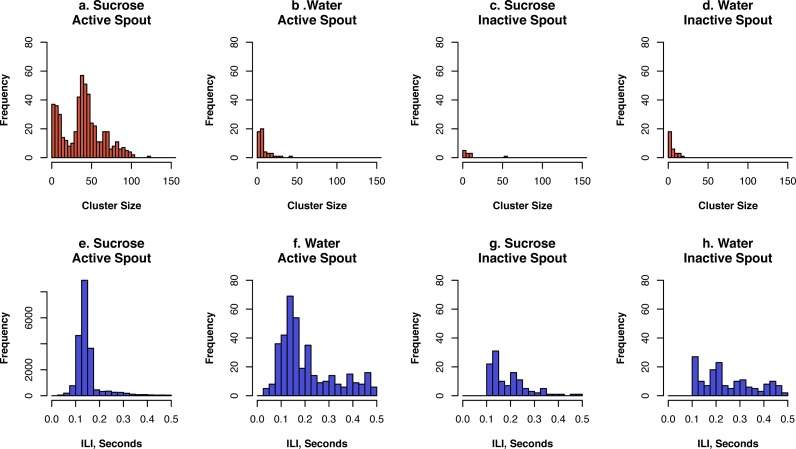
Lick microstructure analysis of the FR10 data set. We defined a cluster as a group of licks where the inter-lick interval (ILI) was less than 0.5 s. Data from all five rats were combined for the analysis.

The microstructure of licks is informative for the reward value of the taste substance (Davis & Smith, 1992). As previously reported (Davis & Smith, 1992; Wang, Wang & Chen, 2014), we define as a cluster those licks that occurred within 0.5 s of each other. Clusters with fewer than two licks were excluded from the analysis. The size of a lick cluster is the number of licks it contains. Inter-licking interval is defined as the time between each lick within each cluster. The distributions of inter-lick intervals and the size of lick clusters for sucrose or water under the FR10 schedule are shown in Fig. 5. The average size of lick clusters on the active spout for sucrose was 35.7 ± 6.4 for sucrose and 7.2 ± 1.1 for water (*p* < 0.05). In contrast, the average size of lick cluster on the inactive spout was 8.6 ± 4.0 for sucrose and 5.9 ± 0.4 for water (*p* > 0.05).

The inter-lick interval on the active spout was 0.15 ± 0.002 s for sucrose and 0.22 ± 0.01 s for water (*p* < 0.01). The inter-lick interval on the inactive spout was 0.20 ± 0.01 s for sucrose and 0.26 ± 0.02 for water (*p* < 0.01).

**Figure 6:**
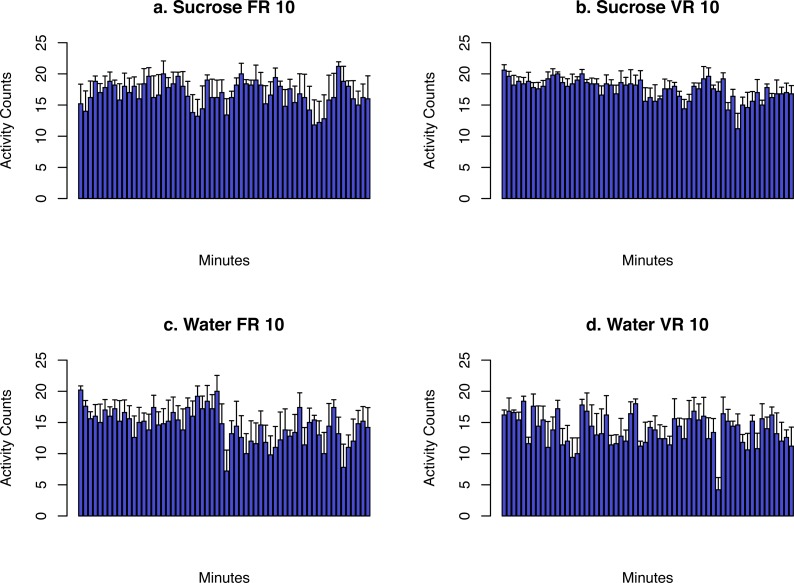
The activity data from each rat were combined into 1 min bins. Rats remained active during the entire 60 min when sucrose was provided, while fewer activity counts were recorded when water was provided.

The activity data collected from the FR10 and VR10 session are presented in Fig. 6. Data from the PR session was not shown because each rat had a different session length. The movement was combined into 1 min bins. The average number within each bin from all the rats were shown. These data showed that although there was a general trend of reducing activity, rats remained active during the entire 1 h session when sucrose was provided. However, the rats were less active when water was provided.

### Environmental data

We tested the reliability of the reading by placing two devices assembled several month apart side by side. Analysing data collected over 200 consequtive time points (10 min interval) showed that the differences between the two devices on temperature, humidity, barometric pressure, and light levels were 0.29 ± 0.35 °C, 1.6 ± 7.7 %, 0.98 ± 0.43 kPa, and 57.6 ± 94.3 Lux, respectively (mean ± standard deviation). The temperature, humidity and barometric pressure readings were in agreement with a comercial instrument. The Lux reading was very sensitive to the angle of the sensor. Because we were using the Lux reading to monitor light cycle (i.e., on/off events), we did not further calibrate this sensor.

**Figure 7:**
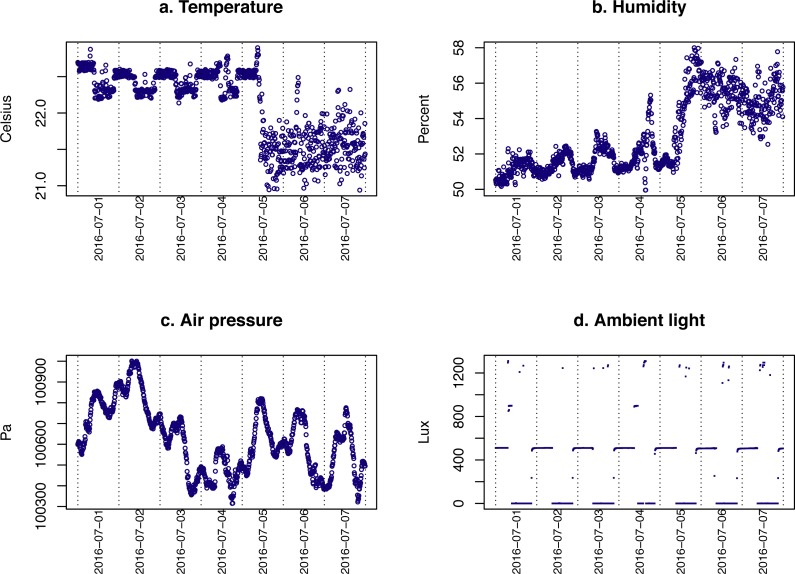
Environmental data. The temperature, humidity, air pressure and level of light in an animal housing room are shown. The shift in temperature and humidity on July 5th coincided with an adjustment of the airflow in the facility.

The data gathered by the environment sensor set from an animal housing room for the first week of July 2016 is plotted in Fig. 7. The data show that the animal facility was under tight climate control. However, after adjusting the airflow on July 5th, the temperature was reduced by ∼1 °C and the humidity was increased by 3%. The air pressure also showed fluctuation. The level of light recorded indicates that the light of the room was reverse cycled (On at 9 PM and off at 9 AM, and that technicians occasionally entered the room during the day.

## Discussion

We designed two devices using the Raspberry Pi computers. The operant licking device cost less than $200 and could be assembled in 3–4 h. We tested the operant licking device by training rats to obtain sucrose or water using several reinforcement schedules in regular housing cages. We also designed a device that can continuously record four environmental variables, including temperature, humidity, air pressure, and light levels. This device cost less than $100 and could be assembled in 1–2 h.

Many behavioral researchers have discovered the relevance of these small computers for data collection projects and taken full advantage of their usefulness. For example, Escobar & Pérez-Herrera (2015) described the use of an Arduino microcontroller in conjunction with a laptop computer to conduct operant conditioning experiments (Thingiverse IDs: 1226076 and 1269418). Pineño (2014) reported an operant conditioning device using an iPod Touch and an Arduino microcontroller. The Kravitz group published two Arduino-based devices. The FED is a home cage-compatible device for measuring feeding behavior in mice (Nguyen et al., 2016), and the ROBucket is a flexible operant chamber (Devarakonda, Nguyen & Kravitz, 2016). Most interestingly, Rizzi, Lodge & Tan (2016) designed an Arduino-based system that triggered the delivery of optogenetic stimulation from nose pokes of mice.

We chose to use single-board computers over microcontrollers because they have faster processors, easy access to permanent data storage, network access, and run on modern operating systems while still being very affordable, although there are situations where microcontrollers have advantages. There are numerous single-board computers available, we chose the Raspberry Pi ($35) because it has a large user community and is the most likely to have sustained development, as demonstrated by the recent release of Raspberry 3, which includes a built-in WiFi module.

Our devices demonstrated the utility of RPi in studying rodent behaviors. The small size of the RPi allows the entire device to fit in a regular rat housing cage. Combined with low cost, this has the potential for breaking down two of the main barriers for many academic labs to conduct operant behavioral tests: the lack of funding for expensive equipment and limited space in the vivarium. Another advantage of shrinking the size of behavioral test equipment is that it allows rodent behavior to be recorded continuously in the home cage. Thus, the diurnal rhythm of the behavior can be recorded without incurring large costs for dedicated equipment.

RFID tags provide unique identification codes for animals. The low frequency (125 kHz) glass tags (∼$1 each) usually encode a twelve character hexdecimal number and therefore can uniquely identify 2.8 × 10^14^ animals for a research project. Because of their small size, they can be inserted into a syringe needle and be injected under the skin of rodents without general anesthesia. Once embedded, they provide a permanent ID for each individual. These tags can even be placed with tissue samples once the animals are euthanized.

Integrating an RFID reader with the behavioral device allows each test subject to be unequivocally identified in the data files. Further, the device can be programmed to start the test session when an RFID is detected. This simplifies the workflow and reduces potential noise in the data. In addition, we also use the RFID system to select the reinforcement schedule. While the program defaults to use the variable ratio 10 schedule, we encoded the value of several RFID tags in the program so that when these particular tags are detected, a fixed ratio or a progressive ratio schedules will be used. Although this is very simple to use and can be readily expanded to include other options, one shortcoming is that we need to use those particular tags to start the program. We remediated this by programming two alternative tags for each reinforcement schedule.

Another potential advantage of the RFID tags is that they allow for the possibility of multiple animals to be tested in a group housing setup. However, in our tests, we have found that the detection of RFID tags using the RDM6300 reader is not sufficiently reliable. It sometimes misses the tag even when the antenna is in close proximity to the tag (maximum sensitivity is found when the tag approaches the antenna in a perpendicular direction and at the edge of the antenna loop. One of the main future directions of this project is to improve the sensitivity of the RFID detection system, possibly by using a different RFID reader, such as those used by Howerton, Garner & Mench (2012).

There are several commonly used methods for recording the licking behavior of rodents. The contact lickometer supplies a small voltage between the wire floor and the spout. It then detects the small current passing through the rat when it licks the spout. This requires a metal floor to be used. An alternative method is to set up an infrared beam to monitor the tip of the spout. The tongue blocks the light beam and allows the licks to be detected. This method requires the position of the light and the spout to be carefully calibrated.

We used a capacitive touch sensor to monitor the licking events. Our analysis of the lick microstructure for sucrose was in agreement with those reported in the literature (Davies et al., 2015), indicating that this device has sufficient time resolution and sensitivity to accurately measure the rapid licking behavior of rats. One caveat is that these touch sensors are very sensitive. They can sometimes be triggered by environmental interference, especially when connected to a large piece of metal, such as a rodent drinking spout. Although we found a sensitivity setting (see ‘Methods’) that was sufficient to avoid the noise while still reliably recorded the licking events, this setting might need to be validated for each type of spouts.

One of the main motivations in developing these devices is to study operant alcohol self-administration in rats. Rats have many advantages for behavioral neuroscience research (Parker et al., 2013). However, many of the widely used rat strains do not readily consume alcohol. We hope the device described here will be helpful in establishing a robust model of oral alcohol self-administration. Operant licking provides potential advantages over lever pressing behavior, such as its high response-reinforcer contingency. Further, the lick microstructure analysis can provide insights into the subjective value of the reward (Davis & Smith, 1992). The combination of low cost and small footprint of this device also provides a unique opportunity to perform relatively large-scale studies on the diurnal rhythm of alcohol consumption.

Lastly, the environmental monitoring device allows the collection of several variables that could potentially influence alcohol intake in the long run and improve experimental reproducibility. Although commonly ignored, more and more recent data show that environmental factors such as temperature (Chesler et al., 2002), humidity (Chesler et al., 2002), barometric pressure (Mizoguchi et al., 2011), noise (Okada et al., 1988; Prior, 2002), illumination (Valdar et al., 2006), and vibration (Okada et al., 1988) have a great impact on animal behavior. Our low cost system can capture large amounts of environmental data, which can improve understanding of the complex effects of the environment on behavior and increase research reproducibility (Collins & Tabak, 2014).

There are several potential disadvantages of our approach comparing to existing commercial products. For example, manufacturing these devices may require skills that are not commonly present in a behavioral neuroscience lab. Although 3D printers have become very affordable, printing high-quality parts is still a trial and error process. High precision is especially important for parts used in the syringe pump. In addition to adjusting the printing condition (e.g., temperature and/or resolution), the design might also need to be modified after the initial printing runs. Because the device is placed in the cage, it has the potential to be chewed by the rats. However, in our daily testing of six devices over three months, this was very rare. Should that become a concern, it is possible to replace just the bottom part of the printed frame, which is most likely to be damaged by rats. Installing the software requires some knowledge of the Linux operating system, especially when a secured WiFi network is used for data transfer (example configuration files for the eduroam network widely used across higher educational institutes, as well as for transferring data are provided in our GitHub repository). Alternatively, the storage media can be taken out via a dedicated slot for copying the data manually. Lastly, because there is no commercial support, trouble shooting may be difficult until there is a community of users. In general, making sure the connections are correct and testing the various components individually (with a keyboard and a monitor connected) would reveal the causes of the problems.

In summary, we developed two open source devices that can be used to collect multi-dimensional data for behavioral studies. By providing the design and software under an open source license, we hope they will stimulate the wider adoption of single-board computers and innovation in behavioral measurements.

##  Supplemental Information

10.7717/peerj.2981/supp-1 Supplemental Information 1 Supplemental Files The complete raw data and several R scripts that generated all the figures and statistical analysis were compressed using the Linux tar command. The bash program dataSummary.sh located in the sucrose and water subdirectory was used to generate summary statistics for each run.

## Additional Information and Declarations

### Data Availability

The following information was supplied regarding data availability: Openbehavior: https://github.com/chen42/openbehavior.
